# Process Evaluation of an eHealth Intervention (Food4toddlers) to Improve Toddlers' Diet: Randomized Controlled Trial

**DOI:** 10.2196/18171

**Published:** 2020-07-06

**Authors:** Margrethe Røed, Frøydis Nordgård Vik, Elisabet Rudjord Hillesund, Wendy Van Lippevelde, Anine Christine Medin, Nina Cecilie Øverby

**Affiliations:** 1 Department of Nutrition and Public Health Faculty of Health and Sports Sciences University of Agder Kristiansand Norway; 2 Department of Marketing, Innovation and Organisation Ghent University Ghent Belgium

**Keywords:** toddler, mHealth, usability, eHealth, diet intervention, digital intervention, education difference

## Abstract

**Background:**

Parents seek trustworthy information online to promote healthy eating for their toddlers. Such information must be perceived as relevant and easy to implement and use.

**Objective:**

The objectives of this study were to conduct a process evaluation of the electronic health (eHealth) intervention (Food4toddlers) targeting food environment, parental feeding practices, and toddlers’ diet and to examine possible differences in these areas according to education and family composition.

**Methods:**

A 2-armed randomized controlled trial, including 298 parent–toddler dyads from Norway, was conducted in 2017. In total, 148 parents in the intervention group received access to an intervention website for 6 months. Data on website usage were retrieved from the learning management platform used (NEO). Participants’ satisfaction with the intervention was asked for in a postintervention questionnaire. Chi-square and *t* tests were used to examine differences in usage and satisfaction between education and family composition groups.

**Results:**

Most participants were mothers (144/148, 97.2%), lived in two-adult households (148/148, 100%), and were born in Norway (132/148, 89.1%). Mean parental age was 31.5 years (SD 4.2). More than 87.8% (129/147) had a university education degree and 56.5% (83/147) had over 4 years of university education. Most (128/148, 86.5%) intervention participants entered the website at least once (mean days of access 7.4 [SD 7.1]). Most parents reported the website as appropriate to the child’s age (71/83, 86%) and self-explanatory (79/83, 95%) and appreciated the interface (52/83, 63%) and layout (46/83, 55%). In total, 61% (51/83) stated that they learned something new from the intervention. Parents with over 4 years of university education and in 1-child households used the intervention website more than those with 4 years or less of university education (8.4 vs 5.9 days in total, *P*=.04) and households with more than 1 child (8.3 vs 5.8 days in total, *P*=.04), respectively.

**Conclusions:**

The Food4toddlers intervention website was found to be relevant by most participants in the intervention group, although usage of the website differed according to educational level and family composition. For eHealth interventions to be effective, intervention materials such as websites must be used by the target group. Our results highlight the need to include users from different groups when developing interventions.

**Trial Registration:**

ISRCTN Registry ISRCTN92980420; http://www.isrctn.com/ISRCTN92980420

## Introduction

A healthy diet is fundamental to preschoolers’ health and development, for which parents are responsible. A high proportion of parents feel insecure and seek advice regarding food parenting practices via different sources [1]. Internet is a powerful and popular source for health information among parents [2-4]. Still, very few theory- and evidence-based websites or digital apps with trustworthy information exist for this group. Among the few electronic health (eHealth) interventions addressing food parenting practices and child diet that have been developed [5-7], most have been conducted in children older than 1 year of age [5]. Furthermore, interventions targeting parents of preschoolers have shown divergent effectiveness [8].

Mobile health (mHealth) and eHealth interventions are gaining popularity, as such interventions have the potential to reach a large target group, can easily be adapted to new groups, are available 24/7, and can be cost-effective [8-10]. However, for eHealth interventions targeting parents of preschoolers to be effective, one needs to take the interplay between parents’ needs and the eHealth intervention’s content into account. This means that the information provided has to fit with the child’s age, be relevant, be easily accessible by the parents, and be perceived as engaging and meaningful [9]. Although the usage and parental satisfaction of eHealth interventions are crucial, little attention has been given to process evaluation of eHealth interventions targeting parents of young children, addressing intervention use and parental intervention satisfaction.

A few other studies have reported on parental use and satisfaction of eHealth interventions targeting young children. One is the Early Food for Future health study, in which Helle et al [11] found that a high proportion of parents used the intervention website and were well satisfied. A recent paper from the Growing Healthy Program in Australia reported both quantitative and interview data on how parents used and whether they were satisfied with an infant health app, concerning mode of delivery and how the quality of the app was perceived [12]. They found that factors such as previous knowledge and parity affected how the participants appreciated the app. This highlights the need for identifying whether there are differences in the use and satisfaction with the app according to group characteristics. Within public health, there is a focus on socioeconomic differences in health and how to reduce this gap [13]. eHealth interventions aim to improve health and should, ideally, work equally well in different socioeconomic groups, meaning that use and perceived satisfaction should be similar in different socioeconomic groups, including in groups with different educational levels.

We have previously developed and evaluated the effect of a dietary eHealth intervention called Food4toddlers in a randomized controlled trial, targeting parents of 12-18-month-old children [14]. The objectives of this study were to conduct a process evaluation of this eHealth intervention by examining the usage and perceived satisfaction of the intervention website among parents of toddlers and to explore whether this differed according to educational level and number of children in the household.

## Methods

### Study Design

Food4toddlers is a randomized controlled trial, aiming to promote healthy dietary habits among toddlers [14]. A total of 404 parents of 12-month-old children were recruited through a Facebook advertisement, who then responded to a baseline questionnaire and were randomized into an intervention group and a control group. Participants in the intervention group were given access to the Food4toddlers website for 6 months. Further, they responded to questionnaires immediately after the end of the intervention (follow-up 1) that included process evaluation measurements, and again 6 months postintervention (follow-up 2).

Eligible individuals were parents of children born between June 2016 and May 2017. The parents had to be literate in Norwegian. Of the 404 recruited parents, 298 (73.8%) filled in more than half of the baseline questionnaire which was the minimum requirement to be randomized into either the control or intervention group (n=148). Postintervention, at child age 18 months (follow-up 1), 220 participants completed all or parts of the questionnaire, with 99 of these from the intervention group. Details of the recruitment strategy, the development of the intervention, and the randomized trial are described in the study protocol [14]. The study was approved by the Norwegian Centre for Research Data on June 08, 2016 (reference number 48643). Informed consent from parents was obtained when they signed in online for participation. Data from the intervention group at baseline and follow-up 1 are reported in this study.

### The Food4toddlers eHealth Intervention

The intervention group had 6 months of access to the Food4toddlers website which comprised 4 main elements: (1) lessons (n=22) on how to provide healthy food and create a healthy eating environment for the toddler, (2) recipes, (3) a discussion forum, and (4) basic information about food and beverages (called *Good to know*). Initially, the web page was limited to information relevant for the child’s age at baseline and gradually expanded in 20 steps as the child got older. The participants received a weekly email with a link to the newly available information. Each module had elements of activities, such as quizzes, videos to watch, facts, and myth busting [14].

### Data and Measurements

In this paper, we present the following elements from the process evaluation: (1) the exposure or usage of the intervention, (2) parental satisfaction with the intervention, and (3) parental perception of learning something new from the intervention. To assess the exposure or usage of the website we used data automatically registered by the Learning Management System NEO. NEO is a platform for managing digital classroom activities and tracking student achievement. It has an intuitive design, making it easy to obtain access to information. The user data were manually retrieved from NEO. The data accessible were (1) number of days the participants accessed the website, (2) the use of the 22 *Food4toddlers* lessons, and (3) activity on a discussion forum. No data on the use of the recipes and the *Good to know* section were available. Some participants visited the website but had no reports on the use of any lessons. They were coded as *1-day users* because they theoretically could have used the rest of the website except the lessons (eg, recipes).

In addition to the automatically registered information on participant’s use of the website, we used data from the postintervention questionnaires. The intervention group responded to questions about the use and satisfaction of the intervention’s website at follow-up 1 (end of intervention). Parents were asked how many of the recipes they had tried, with response alternatives *none*; *none, but was inspired*; *1-5*; *6-10*; and *11 or more.* We further asked them which part of the intervention they found most useful (lessons, recipes, *Good to know* site, or whether they did not know what they preferred). Further, the parents graded statements about their satisfaction (1-7) with the intervention and perception of learning something new (8): Do you agree or disagree with these statements: *(1) The content was well adapted to my child's age*, *(2) The text was understandable, (3) The website was user-friendly, (4)* *The website had an appealing layout, (5) The recipes were easy to follow, (6) The recipes were easily adapted for the whole family, (7) The films for the recipes were useful,* and *(8) I learned something new.* The response alternatives were given by a 5-point scale from 1=*strongly disagree* to 5=*strongly agree* with an additional *I don’t know* response alternative. The answers were recoded into 3 groups for the analyses in this paper (*agree, indifferent,* or *disagree*).

### Other Measures

Parents’ height and weight were self-reported. For their child, measures recorded at the health care centers were reported if available. The participants reported their age and their child’s age at baseline. Further, they reported the number of persons in the household in 2 different questions: (1) number of adults and (2) number of children. They also reported county of residence and marital status (married, partnered, single, divorced/separated, widow/er, or other). The number of children in the household was dichotomized into those with 1-child households and those with more than 1 child in the household. Participants also reported on their level of education (primary school or less, primary schools plus 1 year of further education, high school, vocational school, upper secondary school or less, college/university [≤4 years], college/university [>4 years], other, and do not know). Only 18 persons were categorized with no higher education, which is a low number when doing subanalyses; therefore, we dichotomized the education variable as presented above. Consequently, the comparisons in this study were between parents with more than 4 years and those with 4 years or less of education, and between parents with 1-child households and those with more children in the household.

### Statistical Analysis

Means with standard deviations for continuous variables and frequencies and percentages for categorical variables were reported. The chi-square tests were used to test potential differences in the perceived value of the intervention between the 2 education groups and according to the number of children in the household. Independent sample *t* tests were used to test potential group differences for continuous variables. All analyses were conducted in SPSS version 25 (IBM). Statistical significance was set to the *P*≤.05 level.

### Availability of Data and Materials

The data set supporting the conclusions of this article will be available in the UiA Open Research repository.

## Results

### Participant Characteristics

The characteristics of the participants included in the intervention are summarized in Table 1. Mean parental age was 31.5 years (SD 4.2; Table 1). Most participants were mothers (144/148, 97.2%), lived in 2-adult households (148/148, 100%), and were born in Norway (132/148, 89.1%). There were participants from all over Norway, originally reported by county of residence, with representation from all 19 Norwegian counties; however, these data are presented in Table 1 as numbers from each of the main parts of Norway. Of the participants in the intervention group, 56.4% (83/147) had more than 4 years of university education.

**Table 1:** Baseline characteristics of parents and toddlers in the intervention group (N=148).

Characteristic		Intervention group
**Parent**		
	Mother/father (n)	144/4
	Age (year), mean (SD)	31.5 (4.4)^a^
	Height (cm), mean (SD)	169 (6.0)
	Weight (kg), mean (SD)	70.8 (14.3)
	BMI (kg/m^2^), mean (SD)	24.9 (4.6)
	Two-adult household^b^, n (%)	148 (100)
	Total number of household members, mean (SD)	3.6 (1.0)
	Born in Norway, n (%)	132 (89.1)
**Educational level^a^**		
	Less than college/university (≤4 years), n (%)	64 (43.5)
	College/university (>4 years), n (%)	83 (56.4)
**Geographic residence**		
	Northern Norway, n (%)	8 (5.4)
	Central Norway, n (%)	16 (10.8)
	Western Norway, n (%)	34 (22.9)
	Southern Norway, n (%)	24 (16.2)
	Eastern Norway (including Oslo), n (%)	66 (44.5)
**Toddlers**		
	Age (months), mean (SD)	10.9 (1.3)
	Child’s sex: Female, n (%)	69 (46.6)

^a^One missing case in this variable.

^b^Live together with the other parent.

### Participants’ Use of the Intervention (Usage)

All 148 persons in the intervention group were included in the analyses based on data retrieved from NEO, including 1 person that first got access to the intervention and then decided to quit and 2 participants that did not get access mistakenly (all 3 with no access data). From the NEO data we found that 13.5% (20/148) of parents in the intervention group did not enter the website at any point (Table 2). The mean number of days of access was 7.4 (SD 7.1). Each of the 22 lessons comprised more than 1 webpage and we registered whether the participants had completed the entire lesson or not. On average, the participants completed 8 of 22 lessons (range 0-22; Table 2).

In the intervention group, 99/148 (66.9%) participants answered at least parts of the questionnaire at follow-up 1. However, only 83/148 (56.1%) participants answered the last questions in the questionnaire that concerned the website use. When evaluating the use of the individual components on the website, most participants in the intervention group reported having used *1-5 recipes* (38/83, 46%) or *none but was inspired* (27/83, 33%; Table 2).

**Table 2:** Participants’ use of the intervention website and recipes tried.

Intervention use^a^		Value
**Website use (N=148)**		
	Did not enter, n (%)	20 (13.5)
	Days of access, mean (SD); min-max	7.4 (7.1); 0-32
	Finalized lessons, mean (SD); min-max	8.0 (7.6); 0-22
**Recipes (number) tried (N=83)^b^**		
	None, n (%)	8 (10)
	None, but was inspired, n (%)	27 (33)
	1-5, n (%)	38 (46)
	6-10, n (%)	9 (11)
	11 or more, n (%)	1 (1)

^a^Data were retrieved from the Food4toddlers website. One participant got access to the intervention but decided to quit. Two did not get access to the intervention mistakenly. These 3 are included in the reported numbers.

^b^Questions answered at follow-up 1 (postintervention at child age 18 months).

### Use of the Intervention Website According to Parental Education and Number of Children in the Household

Participants with more than 4 years of university education accessed the website for significantly more days than those with a lower educational level (*P*=.04). In addition, those with more than 4 years of university education completed more lessons than those with fewer years of education (*P*<.05). There was also a difference in use between parents living in 1-child households and those living in a household with more than 1 child. Parents in 1-child households accessed the website for significantly more days compared to those with more children (*P*=.04; Table 3).

**Table 3:** Comparison of website use between education groups (N=147) and between 1-child and >1 child households (N=148).

Analyzed component	≤4 years of university education^a^ (N=64)	>4 years of university education^a^ (N=83)	*P* value^b^	Household with 1 child^c^ (N=86)	Household with >1 child^c^ (N=62)	*P* value^b^
Days of access in total, mean (SD)	5.9 (6.8)	8.4 (7.2)	.04	8.3 (7.8)	5.8 (5.7)	.04
Number of lessons finished, mean (SD)	6.6 (7.3)	9.1 (7.7)	<.05	8.9 (7.8)	6.7 (7.2)	.09

^a^Parents were divided based on educational level into those with 4 years or less of university education and those with more than 4 years of university education.

^b^Independent sample *t* test.

^c^Asked about how many children were included in the household, divided into 1 child versus more children.

### Satisfaction of the Intervention Website’s Modules and Topics

When asked about what part of the intervention website the participants found to be most useful, 43% (36/83) were most satisfied with the recipes, whereas 31% (26/83) valued the modules as the most useful part of the intervention. Participants also reported to which degree they agreed with different statements regarding how they found the intervention website. The majority of the participants agreed that the website content applied to their child’s age (71/83, 86%) and that the texts were easy to understand (79/83, 95%). Most parents in the intervention group reported that they appreciated the interface (52/83, 63%) and layout (46/83, 55%). We also asked to which degree the participants valued the recipes and films. In total, 83% (62/75) found the recipes easy to follow, and 80% (60/75) found them easy to adjust to the whole family. Only 32% (24/75) found the films posted on the intervention website useful. There were no significant differences in how the intervention website and the recipes were valued between those with more than 4 years of university education and those with a lower educational level (data not shown).

There was low activity in the discussion forum including in the learning platform. The most active participant posed questions and responded 5 times, whereas 7 other participants posed a single question during the period when they had access to the forum. The first author (MR) of this paper responded to all questions.

### Perceived Acquisition of New Knowledge From the Intervention Website According to Educational Level and Number of Children in the Household

In total, 61% (51/83) reported that they learned something new from the intervention website (Table 4). There was a borderline significant difference between educational groups when asked whether the participants had learned something new (*P*=.052). More of the highly educated participants agreed that they had learned something new, whereas more participants with moderate education were indifferent to this statement (Table 4).

**Table 4:** Perceived acquisition of new knowledge among parents in the intervention group according to educational level and number of children in the household, through response to the prompt "Think of the Food4toddlers website in total, and indicate how strongly do you agree/disagree with the statement *I learned something new?*"

Statement	All (N=83)	≤4 years of university education^a^ (N=33)	>4 years of university education^a^ (N=50)	*P* value	One-child household^b^ (N=52)	>1 child in household^b^ (N=31)	*P* value
Agree, n (%)	51 (61)	17 (52)	34 (68)	—^c^	35 (67)	16 (52)	—^c^
Indifferent, n (%)	21 (25)	13 (39)	8 (16)	—^c^	12 (23)	9 (29)	—^c^
Disagree, n (%)	11 (13)	3 (9)	8 (16)	.05	5 (10)	6 (19)	.30

^a^Parents were divided based on educational level into those with 4 years or less of higher-level education and those with more than 4 years of higher-level education.

^b^Parents reported how many children were included in the household, divided into 1 child versus more children.

^c^Not applicable.

## Discussion

### Principal Findings

Most parents today use the internet to obtain information relevant to their child’s health [15]; however, they report that they need more training to distinguish between trustworthy and not trustworthy sources [16]. In the Food4toddlers study, we developed a website with evidence-based information relevant to toddlers’ diet, food environment, and parenting practices. More than 86.5% (128/148) of parents in the intervention group visited the website and most of them found the website useful, especially the modules and the recipes. The website content, texts, and interface were highly valued by most parents, which may have influenced parental engagement on the website. Besides, most parents in the intervention group found the content applicable to their child’s age. This is an important result, as it is established that finding the information presented appropriate and given at the right time are essential to change behavior [9].

Although the participants rated the recipes as the most important part of the intervention, they did not find the films made for the recipes as useful as the other components. This may indicate that written recipes might be sufficient for use, or that our produced films did not quite suit the target group. Few participants used the discussion forum which was a part of the website. It might be that parents discuss in other online forums and that our forum seemed new and different, or of no need. Using a closed Facebook group, which is a common discussion forum type, might have increased the activity in the discussions. This is supported by a study by Boswell and collaborators [17] in which parents reported Facebook as the preferred digital platform for participating in an intervention. However, in the parent-focused Time2bHealthy study closed Facebook groups were made available, but less than 40% agreed or strongly agreed that the Facebook component was useful [18]. Our goal with including such a discussion forum was that participants could motivate each other and share experiences; however, as also others have found [18], the inclusion of such a forum might not be worth the effort of setting up.

A total of 13.5% (20/148) of parents who had access to the intervention website did not enter it at any point, which is higher than what is observed in other studies. The Swedish MINISTOP study had a very high website visitor rate [19], possibly because the investigators met the participants in person and called them on the phone 2 days after log-in instructions were delivered. Although we sent email reminders to the participants who did not log in, the adherence might have been higher by adding, for example, a phone call as in the MINISTOP study. Other studies have also emphasized personal contact (eg, the Australian Time2bHealthy study) [18]. However, the costs rise with more intensive follow-up of participants and will limit distribution to a large population. In addition, the website visitor rate in our study is probably more in line with what can be expected when offering access to a web-based learning tool outside a test situation. Boswell and collaborators [17] interviewed parents about their preferred mode of intervention participation and found that they preferred a combination of online sources (websites, email, or Facebook). Parents with lower education levels also preferred this combination; however, in this group, more parents wanted to combine the online scores with face-to-face components [17]. It is worth noting that the use of more advanced push notifications is increasingly being used in digital health interventions [20,21], and could have boosted both the participation and the parental engagement on the website.

There were differences in website use between education groups and between those with 1 or more children in the household. It is somewhat surprising that those with the highest education spent more time using the website, and also that there is a borderline difference in whether they found that they had learned something new from the website, with results in favor of the more educated parents. Taki and collaborators [12] reported that parents defined as knowledgeable in parenting skills found eHealth interventions less useful because they did not learn anything new from it. Having a higher education does not translate directly into parenting skills, and one could speculate that higher education creates a higher drive to learn more. However, in the light of public health efforts to reduce social differences in health, this finding is not a positive one, as it indicates that interventions of this kind might increase the socioeconomic divide. It is worth noting that the cutoff between education groups in this study was set high, due to the educational characteristics of the sample. The findings of this study may, therefore, indicate that there are differences in the gain of health-related information as well between parents with higher education. Although we included a diversity of user groups in the development phase of the intervention, including mothers of lower socioeconomic group, we could have put even more emphasis to tailor the content and interface to different groups. A pilot study including parents with different socioeconomic groups or parents with different educational levels would probably have given valuable input, especially followed by interviews targeting both high and low adherent participants.

It was not surprising that those with more children in the household, and thereby more experience in feeding toddlers and potentially less time available, spent less time on the intervention than those in 1-child households. This is in line with what Taki et al [12] describe, that is, previously acquired knowledge about infant feeding yields lower engagement in eHealth intervention of that topic.

### Strengths and Limitations

We obtained objective information about parental access to the intervention from the learning management system (NEO). This means we did not need to solely rely on participants’ self-reported responses to the postintervention questions, which is a clear strength of this study. When interpreting the effect results of this intervention, it is a clear strength that a detailed process evaluation has been conducted.

The participants in our study had a substantially higher educational level compared with national figures [22]. This may compromise the generalizability of our findings. A different spread in educational level would probably have yielded different results, as indicated in other studies [23,24]. Our results highlight the importance of working hard to include not just highly educated groups in studies, as is the case with this study. The overall high educational level in this study influenced our educational level cutoff. Further, although participants were from all Norwegian counties, proportionally more participants were from the southern parts compared with national figures [25], which may hamper generalizability.

### Conclusion

Few previous eHealth interventions focusing on diet have reported data from process evaluations, including parental usage and satisfaction with the intervention, as is the case with this study. We found that most participants used the intervention website during the intervention period, and that they found it relevant and useful. Parents with more than 4 years of university education used and learned more from this intervention than those with a lower educational level. Our findings highlight the utmost importance of including users from different groups when developing eHealth interventions and may inform future interventions to take particular care in matching intervention content to different educational and socioeconomic groups’ needs.

## Appendix

Multimedia Appendix 1 CONSORT-eHEALTH checklist (V 1.6.1).

## Abbreviations

eHealth: electronic health
mHealth: mobile health
